# Semaphorin, neuropilin and VEGF expression in glial tumours: SEMA3G, a prognostic marker?

**DOI:** 10.1038/sj.bjc.6604641

**Published:** 2008-09-09

**Authors:** L Karayan-Tapon, M Wager, J Guilhot, P Levillain, C Marquant, J Clarhaut, V Potiron, J Roche

**Affiliations:** 1 Université de Poitiers, EA 3805, CHU de Poitiers, 2 rue de la Milétrie, BP 577, Poitiers F-86021, France; 2 Université de Poitiers, CHU de Poitiers, Service de Neurochirurgie, 2 rue de la Milétrie, BP 577, Poitiers F-86021, France; 3 Université de Poitiers, CHU de Poitiers, Clinical Investigation Center, CIC INSERM 802, 2 rue de la Milétrie, BP 577, Poitiers F-86021, France; 4 Université de Poitiers, CHU de Poitiers, Laboratoire d'Anatomo-Pathologie, 2 rue de la Milétrie, BP 577, Poitiers F-86021, France; 5 Institut de Physiologie et Biologie Cellulaires, CNRS UMR 6187, Université de Poitiers, 40 avenue du Recteur Pineau, Poitiers F-86022, France

**Keywords:** adult gliomas, semaphorin, neuropilin, VEGF

## Abstract

Gliomas are characterised by local infiltration, migration of tumour cells across long distances and sustained angiogenesis; therefore, proteins involved in these processes are most likely important. Such candidates are semaphorins involved in axon guidance and cell migration. In addition, semaphorins regulate tumour progression and angiogenesis. For cell signalling, class-4 semaphorins bind directly to plexins, whereas class-3 semaphorins require additional neuropilin (NRP) receptors that also bind VEGF_165_. The anti-angiogenic activity of class-3 semaphorins can be explained by competition with VEGF_165_ for NRP binding. In this study, we analysed the expressions of seven semaphorins of class-3, SEMA4D, VEGF and the NRP1 and NRP2 receptors in 38 adult glial tumours. In these tumours, SEMA3B, SEMA3G and NRP2 expressions were related to prolonged survival. In addition, SEMA3D expression was reduced in high-grade as compared with low-grade gliomas. In contrast, VEGF correlated with higher grade and poor survival. Thus, our data suggest a function for a subset of class-3 semaphorins as inhibitors of tumour progression, and the prognostic value of the VEGF/SEMA3 balance in adult gliomas. Moreover, in multivariate analysis, SEMA3G was found to be the only significant prognostic marker.

Gliomas are the most frequent human primary brain tumours. Malignant gliomas are characterised by local infiltration and migration of tumour cells across long distances and, unlike other types of cancer, the morbidity and mortality from most brain tumours do not involve metastasis but rather local invasion of the tumour preventing complete surgical resection. Today, the current standard of care for patients with high-grade malignant gliomas includes tumour resection followed by adjuvant radiotherapy (RT) and chemotherapy (CT). However, the prolongation of survival associated with CT is only equivalent to an absolute increase in 1-year survival of 6% (from 40 to 46%; Stewart, 2002). Recently, a survival benefit was reported for glioblastoma patients treated with temozolomide combined with RT (Stupp *et al*, 2007). This benefit was significantly observed when the *MGMT* promoter encoding an alkyltransferase is methylated (Hegi *et al*, 2005). Nevertheless, despite advances in surgical and medical neuro-oncology, the prognosis of patients with glioma remains poor.

Molecular biology studies have underscored the function of oncogenes in glioblastoma progression, notably *EGF*, *PDGF* and their receptors, and a variety of tumour suppressor genes, particularly *p16*^*INK4a*^, *p14*^*ARF*^, *PTEN*, *RB1* and *TP53*. Also, frequent loss of heterozygosity at chromosomes 1p, 10p, 10q, 19q and 22q suggests a participation of additional tumour suppressor genes (Kleihues and Cavenee, 2000; Pietsch and Wiestler, 1997; Collins, 2002, 2007). However, the current knowledge of tumour genetics does not always allow identifying clinically relevant factors predictive of outcome or response to therapy. More detailed knowledge of underlying mechanisms and their relevance for the cancer process will allow targeting specific deregulated pathways, leading to rational design of future treatment modalities according to the biology of the individual tumours.

As local invasion and migration of tumour cells are pivotal mechanisms in glioma progression, proteins involved in these processes are most likely important. Such candidates are semaphorins, a family of proteins expressed in the brain and other tissues, that are involved in axon guidance and cell migration. In vertebrates, semaphorins are represented by secreted members (class-3 semaphorins), transmembranous members (classes 4–6) and by one class-7 membrane-anchored protein (Semaphorin Nomenclature Committee, 1999; Yazdani and Terman, 2006). Membranous semaphorins, such as SEMA4D, bind directly to plexins (Tamagnone *et al*, 1999), whereas class-3 semaphorins require additional receptors, NRP1 or NRP2 neuropilins (He and Tessier-Lavigne, 1997; Kolodkin *et al*, 1997). In the central nervous system, class-3 semaphorins mostly act as chemorepulsive cues (Messersmith *et al*, 1995; Adams *et al*, 1997; Bagnard *et al*, 1998). In addition, semaphorins and NRPs regulate a wide range of biological processes, including physiological and tumour angiogenesis, tumour progression, immune response and platelet function.

The involvement of class-3 semaphorins in angiogenesis can be explained by NRP receptors shared between neuronal and endothelial cells (ECs). In ECs, NRP1 enhances the interaction of vascular endothelial growth factor, VEGF_165_, with its receptor VEGFR2 and stimulates VEGF-mediated angiogenesis (Soker *et al*, 1998, 2002), whereas SEMA3s are inhibitory by competing with VEGF_165_ for NRP binding (Miao *et al*, 1999). Thus, the ratio of SEMA3 and VEGF expression levels might be an important determinant for blood vessel and tumour development.

VEGF is a major permeability and pro-angiogenic factor that is highly expressed in brain tumours (Plate *et al*, 1992) and is partly responsible for the loss of the blood–brain barrier (Jain *et al*, 2007). Intra-tumoural levels of VEGF and its receptors were related to the histological grade of gliomas (Schmidt *et al*, 1999) and with tumour vascularity (Samoto *et al*, 1995). Elevated expression of the VEGF_165_ receptor NRP1 was found in tumour cells from various human cancers (Bielenberg and Klagsbrun, 2007).

In glioblastomas, NRP1 expression was detected in ECs of proliferating vessels and in neoplastic astrocytes (Broholm and Laursen, 2004). Moreover, NRP1 overexpression was related to poor prognosis in human gliomas and with the malignancy of astrocytic tumours (Osada *et al*, 2004; Hu *et al*, 2007). Increased expression of NRP1 has also been detected in tumour cells from clinical glioma samples, suggesting a link between NRP1 expression and glioma malignancy (Ding *et al*, 2000). Besides VEGF binding, a novel mechanism was recently proposed for NRP1 to promote tumour progression through the enhancement of autocrine hepatocyte growth factor/scatter factor signalling through c-Met (Hu *et al*, 2007; Matsushita *et al*, 2007). NRP2 could also participate in human glioma progression (Mariani *et al*, 2001).

To date, little is known about the implication of semaphorins in gliomas. In 12 human glioma cell lines, Rieger *et al* (2003) showed that SEMA3A and SEMA3C were always expressed, whereas only some cell lines expressed NRP1, NRP2, plexins A1, A2 or B1.

Recently, on the basis of the Affimetrix gene chip analysis of gliomas, it was shown that SEMA3B expression associated with poorer overall survival (OS) when combined with the expressions of two other genes, osteonectin/SPARC and doublecortex/doublecortin, which have key functions in cellular migration processes (Rich *et al*, 2005). However, *SEMA3B*, like *SEMA3F*, is described as a tumour suppressor gene (Tomizawa *et al*, 2001; Tse *et al*, 2002). *SEMA3F* acts as a tumour suppressor gene by reducing angiogenesis and metastasis, probably through the inhibition of integrin-mediated adhesion and VEGF expression (Xiang *et al*, 2002; Kessler *et al*, 2004; Bielenberg *et al*, 2004; Kusy *et al*, 2005; Futamura *et al*, 2007; Potiron *et al*, 2007). *SEMA3B* and *SEMA3F* are also direct p53 targets (Ochi *et al*, 2002; Futamura *et al*, 2007). In contrast to these inhibitory semaphorins, SEMA3C, 3E, 5C, 6A, 6B may contribute to tumorigenesis or to tumour progression (Neufeld *et al*, 2005). Also, the transmembranous semaphorin SEMA4D has recently become a focus of intensive research owing to its capacity to induce tumour cell invasiveness and angiogenesis (Conrotto *et al*, 2004, 2005; Basile *et al*, 2006, 2007; Ch'ng *et al*, 2007). Thus, the role of semaphorins in human gliomas is ambiguous. Moreover, to our knowledge, SEMA3D, SEMA3E, SEMA3F and SEMA3G expressions in human gliomas were not studied.

In this study, we analysed mRNA expression of class-3 semaphorins, in addition to SEMA4D, VEGF, NRP1 and NRP2 expressions, in 38 adult glial tumours. Statistical analysis indicated that SEMA3B, SEMA3G and NRP2 expressions were related to prolonged survival and that SEMA3D expression was reduced in high-grade as compared with low-grade gliomas. In contrast, VEGF expression was related to higher grade and poor survival. Thus, our data suggest a function for a subset of class-3 semaphorins as inhibitors of tumour progression, and the prognostic value of the VEGF/SEMA3 balance in adult gliomas. In addition, in multivariate analysis, SEMA3G and age were found to be the only significant prognostic markers.

## Patients and methods

### Patients

Tissues from 38 adult patients harbouring glial tumours including 11 low-grade and 27 high-grade gliomas were collected during surgery at the Department of Neurological Surgery (University of Poitiers, France), with signed informed consent of all patients and the approval of the ethics committee of the Poitou-Charentes area. These patients were free from any past medical history, especially with regard to brain surgery, brain radiation therapy or CT. All patients were treated between January 2002 and May 2003. Twenty-two patients had a combined therapy that associated with surgery plus RT and/or CT, whereas 16 patients had no RT. Tumour diagnosis and grading were established according to the World Health Organization (WHO) criteria (Kleihues *et al*, 2002) and were systematically revised by two expert neuropathologists. Distribution of patients in groups according to the WHO pathology classification is described in Table 1.

### Analysis of class-3 semaphorins, VEGF, NRP1 and NRP2 expressions

*Tissue harvesting and preparation* Per-operative pathology exam permitted the checking of glial tumour diagnosis with *s*amples obtained from either open-sky surgery or stereotactic biopsies. Each tumour sample was divided into two parts: one was dedicated to smears and the second was immediately frozen in liquid nitrogen in the operating room, and stored at −80°C until usage. Only samples containing at least 80% of tumour cells were considered for quantitative real-time RT–PCR.

*RNA isolation and cDNA preparation* Total RNA was extracted from 0.5 to 3 mg of tumour tissues using the RNAeasy® Mini Kit (Qiagen, Courtaboeuf, France) and cDNA was prepared as described earlier (Wager *et al*, 2006).

*Quantitative real-time RT–PCR* mRNA levels were measured by quantitative real-time RT–PCR in the ABI PRISM 7000 sequence detection system (Applied Biosystems, Courtaboeuf, France). Primer sequences and the length of the PCR products are listed in Supplementary Table 1. The specific amplification for all transcripts was checked by DNA sequencing after DNA purification from the unique band of the RT–PCR product obtained at the right size by agarose gel electrophoresis and by the thermal dissociation curves (Supplementary Figure S1). Amplification efficiency was tested using serial dilutions of each specific PCR product, and the quality of the amplification curves was similar to the results we described in one of our previous study (Brambilla *et al*, 2000). The quantitative real-time RT–PCR values for each amplification for our cohort of patients are given in Supplementary Table 2. The reactions were carried out by using the SYBR Green chemistry as described earlier (Brambilla *et al*, 2000; Wager *et al*, 2006). The amount of target mRNA was normalised with the endogenous GAPDH mRNA by the [2^−Δ*C*_t_^ × 1000] formula, where Δ*C*_t_ = *C*_t_target__ − *C*_t_GAPDH__. The values of the differences between the *C*_t_ values for GAPDH mRNA and each tested transcript ranged as follows: *C*_t_ SEMA3A−*C*_t_ GAPDH: 5.8–16.7; *C*_t_ SEMA3B−*C*_t_ GAPDH: 5.3–10.1; *C*_t_ SEMA3C−*C*_t_ GAPDH: 5.6–15.4; *C*_t_ SEMA3D−*C*_t_ GAPDH: 7.5–21.6; *C*_t_ SEMA3E−*C*_t_ GAPDH: 2.9–15.0; *C*_t_ SEMA3F−*C*_t_ GAPDH: 4.8–16.8, *C*_t_ SEMA3G−*C*_t_ GAPDH: 7.7–17.4; *C*_t_ SEMA4D−*C*_t_ GAPDH: 6.8–10.8; *C*_t_ VEGF−*C*_t_ GAPDH: 1.8–7.6; *C*_t_ NRP1−*C*_t_ GAPDH: 5.3–10.5; and *C*_t_ NRP2−*C*_t_ GAPDH: 4.0–10.3.

### Statistical analysis

Data were collected from the date of diagnosis. Differences between groups in the respective semaphorin distribution were tested using the Mann–Whitney *U*-test. Correlation analyses were performed by the method of Spearman. Overall survival was analysed by calculating the time interval between the date of diagnosis and the date of death from any cause, or the date of the last follow-up for surviving patients, and then estimated by the Kaplan and Meier method.

The following clinical and biological features were analysed as potential prognostic factors: age, sex, grade (high *vs* low), treatment (surgery and RT±CT *vs* others), semaphorins, NRPs and VEGF. Tested transcripts were categorised in three groups (low, medium and high expressor), such as age (low, medium, high), according to their lower and higher respective quartile values.

All variables were assessed in univariate analysis using the two-tailed log-rank test. To summarise prognostic information, variables found to be associated at the 10% level with the outcome were entered into a Cox regression model on the basis of likelihood ratio test. A stepdown procedure allowed those variables adding to each other's prognostic information to be retained. Levels of significance were represented by *P*-values derived from two-sided tests. A *P*-value <0.05 or less was considered to indicate statistical significance. SAS v 8 (Statistical Analysis System, Cary, NC) software package was used.

## Results

**Figure 1:**
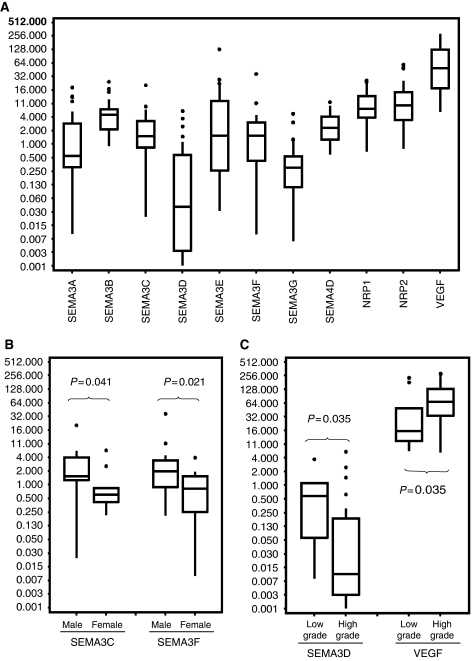
(**A**) Semaphorin, NRP and VEGF expressions in gliomas measured by quantitative real-time RT–PCR; (**B**) SEMA3C and SEMA3F expressions in men and women; (**C**) SEMA3D and VEGF expressions in low- and high-grade gliomas. Results are expressed by [2^−Δ*C*_t_^ × 1000], where Δ*C*_t_ = *C*_t_target__ − *C*_t_GAPDH__.

We studied by quantitative real-time RT–PCR the expression of the seven semaphorins from class-3 (A-G), SEMA4D, NRP1, NRP2 and VEGF in 11 adult low-grade and 27 high-grade gliomas. The largest distribution of the values was observed for SEMA3D, whereas SEMA4D showed a narrower range distribution (Figure 1A). There was no correlation between the expression levels of each tested gene and the age of the patients. Interestingly, SEMA3C and SEMA3F mRNA levels were statistically more expressed in men than in women (*P*=0.041 and *P*=0.021, respectively) (Figure 1B, Supplementary Table 2A).

Between low- and high-grade gliomas, there was a statistically significant difference in SEMA3D and VEGF expressions. SEMA3D was more expressed in low-grade than in high-grade gliomas (*P*=0.035). In contrast, VEGF was more expressed in high-grade than in low-grade gliomas (*P*=0.035) (Figure 1C and Supplementary Table 2B).

We also noticed several correlations between gene expressions (Supplementary Table 3). Correlation between several semaphorin expressions was found positive. The strongest correlation was observed for SEMA4D, which correlated positively with SEMA3B (*P*<10^−4^), SEMA3D (*P*<10^−4^) and SEMA3G (*P*=0.001). SEMA3D correlated positively with SEMA3B (*P*=0.033) and SEMA3G (*P*<10^−3^). NRP1 and NRP2 were also positively correlated (*P*<10^−3^). In addition, SEMA3A and SEMA3B were inversely correlated (*P*=0.007). SEMA3B, SEMA3F and SEMA4D expressions correlated positively with NRP1 (*P*=0.012, *P*=0.007 and *P*=0.009, respectively), whereas SEMA3D and SEMA3G expressions correlated with NRP2 (*P*=0.040 and *P*=0.033, respectively). In contrast, VEGF expression was not correlated with NRPs but was inversely correlated with SEMA3D (*P*<10^−3^), SEMA3G (*P*<10^−4^) and SEMA4D (*P*=0.008) expressions.

**Figure 2:**
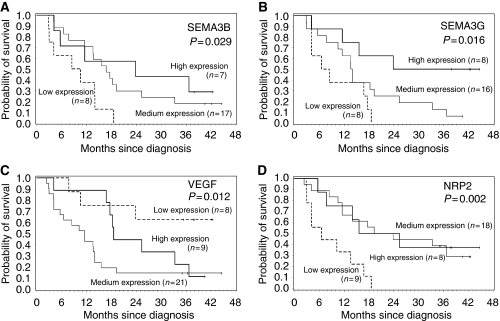
Kaplan–Meier survival curves for 38 patients with glial tumours, for SEMA3B (**A**), SEMA3G (**B**), VEGF (**C**) and NRP2 (**D**) expressions. Three groups of patients were defined as follows: group 1 included patients whose expression for the tested transcript was below the 25th quartile, group 2 for expression included between the 25th and 75th percentile values and group 3 for expression above the 75th quartile.

To determine the clinical relevance of these findings, with regard to patient outcome, we also assessed whether these biomarkers were related to OS. Patients were divided in three groups as follows: group 1 included patients whose expression for the tested transcript was below the 25th quartile, group 2 for expression between the 25th and 75th percentile values and group 3 for expression above the 75th quartile. Results of the univariate analysis are summarised in Table 2A. Among the tested semaphorins, SEMA3B and SEMA3G mRNA levels proved to be a prognostic marker for OS in our cohort including 11 low-grade and 27 high-grade gliomas (*P*=0.029 and *P*=0.016, respectively) (Figure 2A and B). All patients with lower SEMA3B mRNA expression died within 20 months after diagnosis (group 1), whereas a probability of death of 82 and 71% after 40 months was observed in group 2 and 3 patients, respectively. The median OS time for groups 1, 2 and 3 were 9 (95% CI: 3–14), 17 (95% CI: 11–25) and 24 (95% CI: 6–.), respectively. For SEMA3G, all patients from group 1 who expressed lower levels of SEMA3G mRNA died within 20 months, whereas the probability of death was 94 and 50% after 40 months for group 2 and 3 patients, respectively. The median OS times for groups 1, 2 and 3 were 7 (95% CI: 4–18), 14 (95% CI: 11–19), 24 (95% CI: 12–.), respectively.

VEGF expression was also a prognostic marker for OS (*P*=0.012) (Figure 2C). Among group 1 patients who presented the lowest VEGF mRNA levels, 38% died after 36 months. The median OS in this group was not reached (95% CI: 11–.). In contrast, group 2 and 3 patients with higher mRNA expression showed decreased rate of OS (86 and 89% of patients died within 20 months, respectively). The median OS times for groups 2 and 3 were 12 (95% CI: 6–14) and 19 (95% CI: 18–33), respectively.

Lastly, NRP2 mRNA levels could also be considered as a prognostic marker of OS *(P*=0.002) (Figure 2D). All patients with lower NRP2 expression died within 20 months, whereas group 2 and 3 patients with higher NRP2 mRNA expression had prolonged survival (71 and 63% of patients died after 40 months, respectively). The median OS times for groups 1, 2 and 3 were 7 (95% CI: 4–14), 22 (95% CI: 12–.), 20 (95% CI: 8–.), respectively. It can be noted that SEMA3A, SEMA3D and SEMA3E were also found to be associated at a 10% level with the OS.

Among clinical features that were analysed, three of them were statistically related with survival: age, grade (high *vs* low) and treatment (surgery and RT±CT *vs* others). The 10 variables that were identified in univariate analysis as possible prognostic factors (*P*<0.10), namely age, grade, treatment, SEMA3A, SEMA3B, SEMA3D, SEMA3E, SEMA3G, VEGF and NRP2 expressions, were considered for multivariate analysis and entered into a Cox model. Only two variables, age and SEMA3G, were selected by stepwise regression with a *P*-value less than 0.05 (*P*=0.009 and *P*=0.011, respectively) and remained considered as adding to each other's prognostic information. Patients with lower age and a higher expression of SEMA3G had a better prognostic for OS (Table 2B). It can be noted that sex was of no prognostic value by univariate analysis, and when this factor was added into the Cox model for adjustment on baseline characteristics, the results remained strictly similar.

## Discussion

Semaphorins are involved in axon guidance, cell migration, development, immunity, tumorigenesis and they regulate tumoural angiogenesis. To better clarify the potential function of semaphorins in the pathogenesis of adult gliomas characterised by high migration potential, marked angiogenesis with endothelial proliferation, severe hypoxia and tumour necrosis, we analysed by quantitative real-time RT–PCR, the expressions of class-3 semaphorins (A–G) and their receptors NRP1 and NRP2 in 38 adult gliomas. The rational to select the expression of class-3 semaphorins and their NRP receptors is that several of these semaphorins have been involved in tumorigenesis and because NRPs are co-receptors for VEGF. The hypothesis is that SEMA3s are competitive inhibitors of VEGF-NRP binding (Miao *et al*, 1999). It thus seemed reasonable to limit the analysis to SEMA3 and NRP expressions on one hand and to VEGF on the other hand. Other specific SEMA3 functions involve plexins as co-receptors that elicit intracellular signals.

In our study, gene expression was compared between tumours as no normal brain sample was available. Resections from epileptic patients are often considered as normal control. However, altered semaphorin expression and NRPs involvement have been reported in epilepsy (for review see Yaron and Zheng, 2007). In mice, Sema3F is downregulated in an epileptogenic-sensitive mice model but not in a resistant one (Yang *et al*, 2005). These results are supported by previous experiments in rats that develop epilepsy after kainic acid injection with the reduction of Sema3F and Sema3C expressions in the hippocampus (Barnes *et al*, 2003). In another model, during the process of electrically induced epileptogenesis in rats, a transient reduction in Sema3A expression was described and correlated to mossy fibre sprouting believed to have a critical function in the hyperexcitability of the hippocampus in temporal lobe epilepsy of patients (Holtmaat *et al*, 2003). Direct functional evidence for a function of semaphorins and their receptors in epilepsy is supported by Sema3F knockout mice (Sahay *et al*, 2005) and some NRP2 mutant mice (Giger *et al*, 2000; Chen *et al*, 2000) that are more prone to seizures. Therefore, we cannot consider resection pieces of epileptic patients as control for normal tissue.

With these limitations, we observed that class-3 semaphorins (A–G) and their receptors NRP1/NRP2 as well as SEMA4D and VEGF expressions were heterogeneous between samples, which suggest a differential expression of these genes between tumours. Consistent with several data (Jain *et al*, 2007), we found that VEGF was significantly more expressed in high-grade gliomas as compared with low-grade gliomas. In contrast, we observed that SEMA3D was statistically more expressed in low-grade than in high-grade gliomas, suggesting that its loss is involved in tumour progression to high grades. To our knowledge, no study was performed for SEMA3D expression in tumours. For the other studied genes (*SEMA3A*, *B*, *C*, *E*, *F*, *G*, *NRP1*, *NRP2* and *SEMA4D*), we did not observe statistical difference between low- and high-grade gliomas. As normal tissue was not available, we can only speculate that either gene expression is not changed in gliomas as compared with normal tissue or that change in expression is an early event in tumour progression.

An interesting observation was that SEMA3C and SEMA3F were statistically more expressed in glial tumours from men than from women, suggesting a possible hormonal regulation of these genes.

In addition, we observed several correlations between semaphorin expressions but we are unable to explain them. Of particular interest were (i) the positive correlation between SEMA4D and SEMA3B, D, G, and (ii) the negative correlation between VEGF and SEMA3D, G and 4D. For this last point, several studies reported VEGF expression inhibition by semaphorins. For example, when VEGF_165_-stimulated multiple myeloma ECs were exposed to SEMA3A, a time-dependent decrease of endogenous VEGF_165_ mRNA was observed (Vacca *et al*, 2006). Similarly, VEGF in addition to NRP1 was downregulated in *SEMA3B*-transfected ovarian adenocarcinoma cells (Naylor, AACR Meeting 2003, Poster no. 4935). In addition, we showed that SEMA3F protein correlated negatively with VEGF in lung cancer (Brambilla *et al*, 2000), and recently demonstrated that SEMA3F reduced VEGF mRNA level and HIF-1*α* protein level (Potiron *et al*, 2007). Lastly, intraperitoneal administration of recombinant extracellular SEMA6A in mouse inhibited both bFGF/VEGF and tumour cell line-induced neovascularisation (Dhanabal *et al*, 2005). One can speculate that SEMA3D and SEMA3G could have similar properties and behave like anti-angiogenic proteins in gliomas. It can be noted that *SEMA3F*, now recognised as a tumour suppressor gene (Xiang *et al*, 2002; Kessler *et al*, 2004; Bielenberg *et al*, 2004; Kusy *et al*, 2005; Futamura *et al*, 2007) with antiangiogenic (Kessler *et al*, 2004; Futamura *et al*, 2007) and anti-metastatic properties (Bielenberg *et al*, 2004), did not show up as an important gene in our cohort of patients with gliomas. One explanation could be that SEMA3F protein is mostly present in nerve fibres and never detectable in glial cells and blood vessel in the adult human brain (Hirsch *et al*, 1999).

The negative correlation between VEGF and SEMA4D is puzzling as SEMA4D triggers invasive growth of epithelial cells (Giordano *et al*, 2002) and is described as proangiogenic through Met recruitment by plexin B1 (Conrotto *et al*, 2005) and stimulation of Rho-initiated pathways (Basile *et al*, 2004, 2006). However, SEMA4D is rare in B-cell non-Hodgkin's lymphomas, suggesting that its loss might lead to decreased cell adhesion, increased mobility and metastatic potential (Dorfman *et al*, 1998). SEMA4D also exerts important functions in the immune system and could be a guidance signal that directs the recruitment of the immune cells (for review Elhabazi *et al*, 2003). Therefore, the involvement of SEMA4D in immunity during tumour development is not known and SEMA4D function in cancer remains ambiguous.

Of particular interest with regard to patient outcome, we found that higher SEMA3B and SEMA3G expressions were related to better outcome. Our observation is in accordance with the tumour suppressor activity of SEMA3B (Tomizawa *et al*, 2001; Tse *et al*, 2002). On the contrary, it was shown that low SEMA3B expression associated with poorer OS in gliomas, but when associated with expression of two other genes, osteonectin/SPARC and doublecortex/doublecortin (Rich *et al*, 2005). However, the very recent data by Rolny *et al* (2008) suggest a reconsideration of this semaphorin as a multifaceted regulator of cancer progression: SEMA3B inhibited tumour growth in mice but simultaneously and unexpectedly triggered metastasis by activating the signalling kinase p38. Regarding SEMA3G, a recently identified semaphorin (Taniguchi *et al*, 2005), no data have been published to our knowledge about its function in tumours. When a multivariate Cox analysis was performed, SEMA3G was found to be, with the age, the only significant prognostic marker. Our study had some limitations as mRNA expression is not always correlated with protein expression; moreover, an aberrant localisation of the protein impaired its activity. Because there is no commercially available/relevant antibody to confirm the expression pattern of SEMA3G, we cannot correlate SEMA3G mRNA and protein levels.

We also observed that higher NRP2 expression or lower VEGF expressions were related to better outcome. Interestingly, NRP2 is the receptor of SEMA3G (Taniguchi *et al*, 2005). As VEGF_165_ binds to NRP2 (Gluzman-Poltorak *et al*, 2000), competition between SEMA3B/3G and VEGF_165_ for binding to NRP2 might exist in gliomas as demonstrated for SEMA3A and NRP1 in ECs (Miao *et al*, 1999).

We did not find any relation between NRP1 expression and OS in our series. This result was surprising because NRP1 is expressed in many tumours, and in some models, NRP1 has been shown to increase tumorigenicity (Miao *et al*, 2000). NRP1 was also significantly correlated with poor prognosis in non-small-cell lung carcinomas (Kawakami *et al*, 2002), and blocking VEGF and NRP1 significantly increased survival (Pan *et al*, 2007).

In conclusion, SEMA3B, SEMA3G and NRP2 expressions were related to prolonged survival of adult patients with glial tumours. SEMA3D expression was reduced in high-grade as compared with low-grade gliomas and the opposite was seen for VEGF expression. Thus, we propose the involvement of a subset of class-3 semaphorins as inhibitors of glioma progression and suggest that the balance VEGF/SEMA3 might be of prognostic value. SEMA3G was found to be the only significant prognostic marker in gliomas when a multivariate analysis was performed. As very little data have been published with regard to this semaphorin, more studies are necessary to assess its function in tumorigenesis.

## Figures and Tables

**Table 1:** Demographic and pathological characteristics of patients

			**Treatment**			**Sex**	
	***n***	**Pathology**	**Biopsy/surgery +RT±CT**	**Biopsy/surgery ±CT**	**Age (years)**	**F**	**M**
Low grades	11	OAII: 7	3	8	42.5	3	8
		ODII: 4			(35–67)	(27%)	(73%)
							
High grades	27	OAIII: 4					
		ODIII: 5	19	8	60.6	11	16
		GBM: 18			(22–75)	(41%)	(59%)

CT=chemotherapy; F=female; GBM=glioblastoma; II, III=tumour grades according to the World Health Organization; M=male; OA=oligoastrocytoma; OD=oligodendroglioma; RT=radiotherapy.

**Table 2A:** Relationship between factors and overall survival in univariate analysis

**Variable**	**Category**	**Log-rank test (*P*-value)**
Age (years)	<43/43–65/>65	0.001
Sex	Male/female	0.866
Grade	High/low	0.002
Treatment	Surgery and RT±CT *vs* others	0.024
SEMA3A expression	Low/medium/high	0.091
SEMA3B expression	Low/medium/high	0.029
SEMA3C expression	Low/medium/high	0.613
SEMA3D expression	Low/medium/high	0.087
SEMA3E expression	Low/medium/high	0.091
SEMA3F expression	Low/medium/high	0.360
SEMA3G expression	Low/medium/high	0.016
SEMA4D expression	Low/medium/high	0.131
NPR1 expression	Low/medium/high	0.161
NPR2 expression	Low/medium/high	0.002
VEGF expression	Low/medium/high	0.012

CT=chemotherapy; RT=radiotherapy.

Gene expression was measured by quantitative real-time RT–PCR.

**Table 2B:** Relationship between factors and overall survival in multivariate analysis (final Cox model)

**Variable**	**Hazard ratio**	**95% Hazard ratio confidence limits**	***P*-value**
Age	1.062	1.015–1.111	0.009
SEMA3G	0.400	0.198–0.810	0.011

Gene expression was measured by quantitative real-time RT–PCR.
