# Supplementary material for: Semaphorin, neuropilin and VEGF expression in glial tumours: SEMA3G, a prognostic marker?
Source: Br J Cancer. 2008 Sep 9;99(7):1153–60. doi: 10.1038/sj.bjc.6604641 (PMC2567090; doi:10.1038/sj.bjc.6604641)
Supplement: Supplementary Figure [file 6604641x1.ppt]

## Slide 1
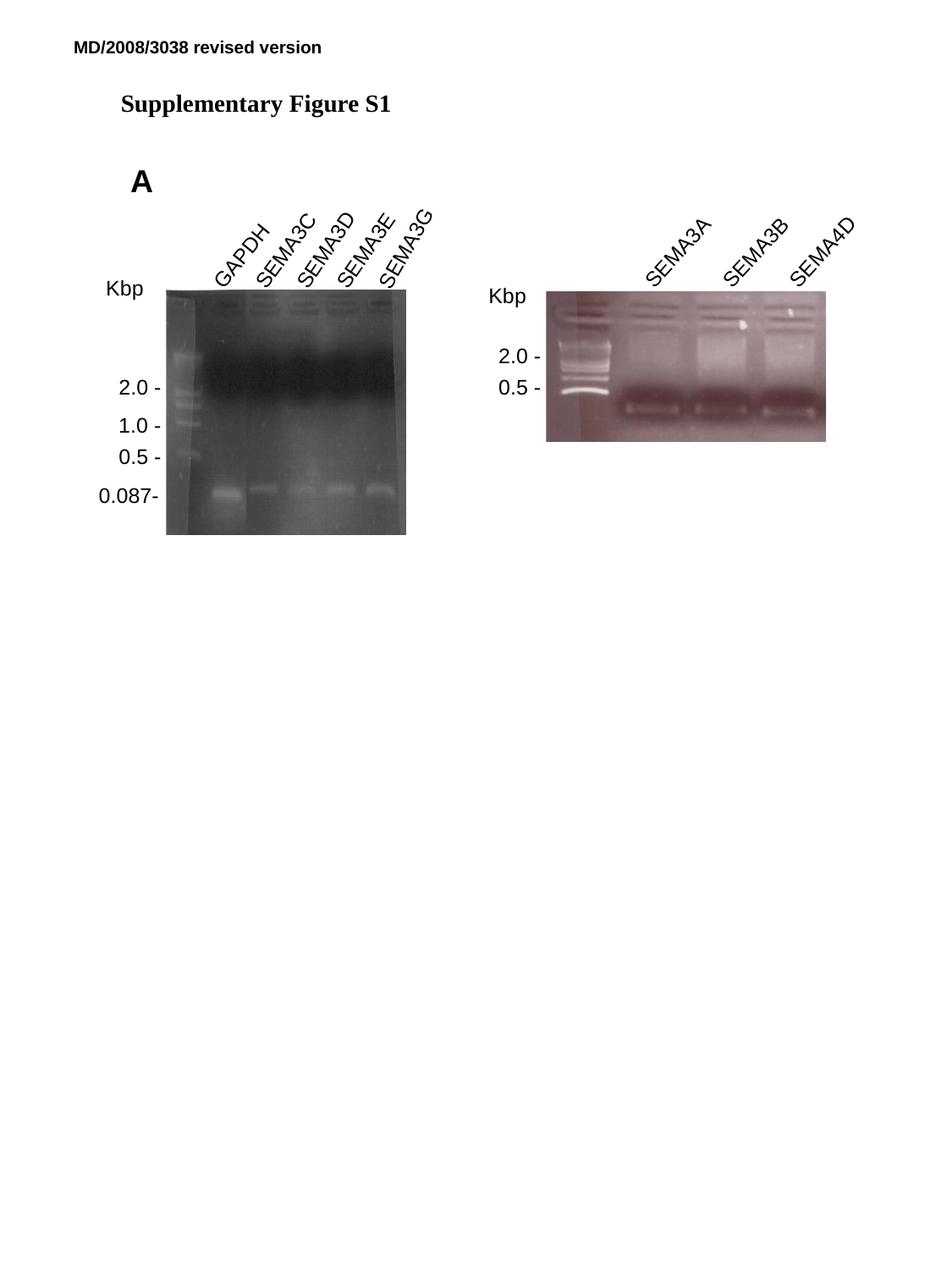

MD/2008/3038 revised version
Supplementary Figure S1
A
SEMA3G
GAPDH
SEMA3D
SEMA3E
SEMA3C
Kbp
2.0 -
1.0 -
0.5 -
0.087-
SEMA4D
SEMA3A
SEMA3B
Kbp
2.0 -
0.5 -

## Slide 2
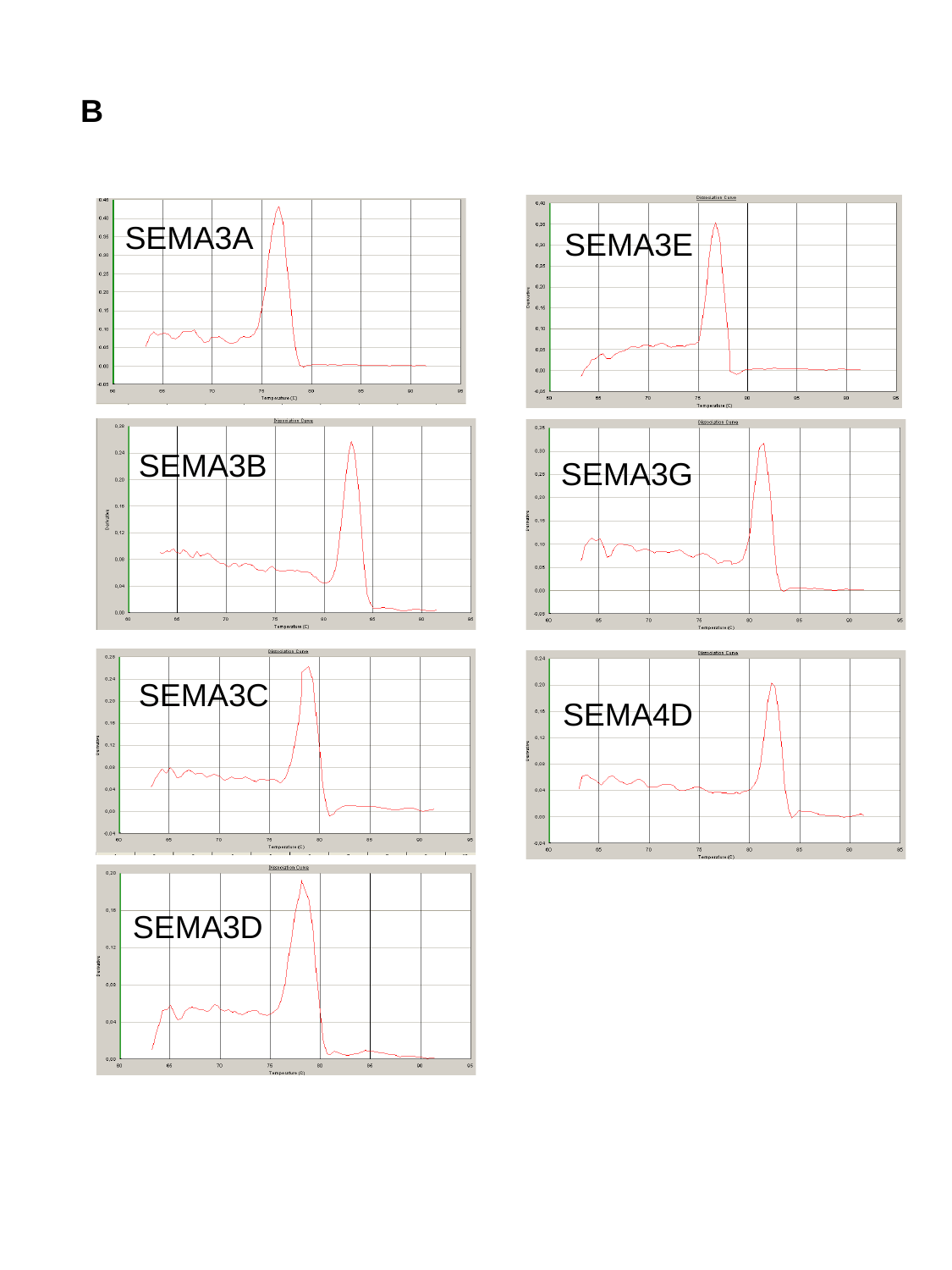

B
SEMA3E
SEMA3G
SEMA4D
SEMA3A
SEMA3B
SEMA3C
SEMA3D
