# Supplementary material for: Semaphorin, neuropilin and VEGF expression in glial tumours: SEMA3G, a prognostic marker?
Source: Br J Cancer. 2008 Sep 9;99(7):1153–60. doi: 10.1038/sj.bjc.6604641 (PMC2567090; doi:10.1038/sj.bjc.6604641)
Supplement: Supplementary Figure [file 6604641x2.doc]

**MD/2008/3038 revised version**

**Supplementary Fig S1**

**C**

**SEMA3B**

CCTTGTTCGGGAGCCTTCACCCAACCTGTGCCTTTGTGGAAGTGGGCCACACAGGCACAGGTTGGGTGGAAGGCTCCAGTGCCACAGGCCAGCAAATGGGTGCGGTTGTAGGCATCAGAAATTA

[ref|NM_004636.2|](http://www.ncbi.nlm.nih.gov/entrez/query.fcgi?cmd=Retrieve&db=Nucleotide&list_uids=54607087&dopt=GenBank&RID=1NGPJERY016&log$=nuclalign&blast_rank=1) [
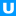
](http://www.ncbi.nlm.nih.gov/entrez/query.fcgi?db=nucleotide&cmd=Display&dopt=nucleotide_unigene&from_uid=54607087&RID=1NGPJERY016&log$=unigenealign&blast_rank=1)[
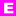
](http://www.ncbi.nlm.nih.gov/entrez/query.fcgi?db=geo&term=54607087%5Bgi%5D&RID=1NGPJERY016&log$=geoalign&blast_rank=1)[
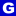
](http://www.ncbi.nlm.nih.gov/entrez/query.fcgi?db=gene&cmd=search&term=54607087%5BNUID%5D&RID=1NGPJERY016&log$=genealign&blast_rank=1)[
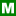
](http://www.ncbi.nlm.nih.gov/mapview/map_search.cgi?direct=on&gbgi=54607087&THE_BLAST_RID=1NGPJERY016&log$=mapalign&blast_rank=1) Homo sapiens sema domain, immunoglobulin domain (Ig), short basic domain, secreted, (semaphorin) 3B (SEMA3B), transcript ,variant 1, mRNA Length=2989, [GENE ID: 7869 SEMA3B](http://www.ncbi.nlm.nih.gov/sites/entrez?db=gene&cmd=search&term=7869&RID=1NGPJERY016&log$=geneexplicitnucl&blast_rank=1)

Score = 117 bits (63), Expect = 2e-24

Identities = 70/73 (95%), Gaps = 1/73 (1%)

Strand=Plus/Minus

Query 47 CCACACAGGCACAGGTTGGGTGGAAGGCTCCAGTGCCACAGGCCAGCAAATGGGTGCGGT 106

||||| ||||||||||||||||||||||||| ||||||||||||||||||||||||||||

Sbjct 672 CCACAAAGGCACAGGTTGGGTGGAAGGCTCCCGTGCCACAGGCCAGCAAATGGGTGCGGT 613

Query 107 TGTAGGCAT-CAG 118

||||||||| |||

Sbjct 612 TGTAGGCATGCAG 600

**SEMA3C**

GATCGGGGTACTGTGCAAAAAGTGGTTGTTCTTCCTACTAACAACTCTG

[ref|NM_006379.2|](http://www.ncbi.nlm.nih.gov/entrez/query.fcgi?cmd=Retrieve&db=Nucleotide&list_uids=32307182&dopt=GenBank&RID=1NH5VSPW01R&log$=nuclalign&blast_rank=1) [
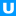
](http://www.ncbi.nlm.nih.gov/entrez/query.fcgi?db=nucleotide&cmd=Display&dopt=nucleotide_unigene&from_uid=32307182&RID=1NH5VSPW01R&log$=unigenealign&blast_rank=1)[
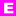
](http://www.ncbi.nlm.nih.gov/entrez/query.fcgi?db=geo&term=32307182%5Bgi%5D&RID=1NH5VSPW01R&log$=geoalign&blast_rank=1)[
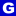
](http://www.ncbi.nlm.nih.gov/entrez/query.fcgi?db=gene&cmd=search&term=32307182%5BNUID%5D&RID=1NH5VSPW01R&log$=genealign&blast_rank=1)[
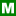
](http://www.ncbi.nlm.nih.gov/mapview/map_search.cgi?direct=on&gbgi=32307182&THE_BLAST_RID=1NH5VSPW01R&log$=mapalign&blast_rank=1) Homo sapiens sema domain, immunoglobulin domain (Ig), short basic domain, secreted, (semaphorin) 3C (SEMA3C), mRNA Length=5189, [GENE ID: 10512 SEMA3C](http://www.ncbi.nlm.nih.gov/sites/entrez?db=gene&cmd=search&term=10512&RID=1NH5VSPW01R&log$=geneexplicitnucl&blast_rank=1)

Score = 97.6 bits (49), Expect = 7e-19

Identities = 49/49 (100%), Gaps = 0/49 (0%)

Strand=Plus/Plus

Query 1 GATCGGGGTACTGTGCAAAAAGTGGTTGTTCTTCCTACTAACAACTCTG 49

|||||||||||||||||||||||||||||||||||||||||||||||||

Sbjct 1916 GATCGGGGTACTGTGCAAAAAGTGGTTGTTCTTCCTACTAACAACTCTG 1964

**SEMA3D**

ATTAAATTGGGGTTTCCGAGATGGATTGGTTCAGCTCTCCTTGCACAGATGCGACACTTATGGGAAAGCTTGCGCAAAAAGGAGAGGA

[ref|NM_152754.2|](http://www.ncbi.nlm.nih.gov/entrez/query.fcgi?cmd=Retrieve&db=Nucleotide&list_uids=41406085&dopt=GenBank&RID=1NHHS057016&log$=nuclalign&blast_rank=1) [
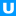
](http://www.ncbi.nlm.nih.gov/entrez/query.fcgi?db=nucleotide&cmd=Display&dopt=nucleotide_unigene&from_uid=41406085&RID=1NHHS057016&log$=unigenealign&blast_rank=1)[
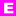
](http://www.ncbi.nlm.nih.gov/entrez/query.fcgi?db=geo&term=41406085%5Bgi%5D&RID=1NHHS057016&log$=geoalign&blast_rank=1)[
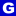
](http://www.ncbi.nlm.nih.gov/entrez/query.fcgi?db=gene&cmd=search&term=41406085%5BNUID%5D&RID=1NHHS057016&log$=genealign&blast_rank=1)[
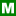
](http://www.ncbi.nlm.nih.gov/mapview/map_search.cgi?direct=on&gbgi=41406085&THE_BLAST_RID=1NHHS057016&log$=mapalign&blast_rank=1) Homo sapiens sema domain, immunoglobulin domain (Ig), short basic domain, secreted, (semaphorin) 3D (SEMA3D), mRNA Length=6276, [GENE ID: 223117 SEMA3D](http://www.ncbi.nlm.nih.gov/sites/entrez?db=gene&cmd=search&term=223117&RID=1NHHS057016&log$=geneexplicitnucl&blast_rank=1)

Score = 117 bits (63), Expect = 2e-24

Identities = 65/66 (98%), Gaps = 0/66 (0%)

Strand=Plus/Plus

Query 11 GGTTTCCGAGATGGATTGGTTCAGCTCTCCTTGCACAGATGCGACACTTATGGGAAAGCT 70

|||| |||||||||||||||||||||||||||||||||||||||||||||||||||||||

Sbjct 1601 GGTTCCCGAGATGGATTGGTTCAGCTCTCCTTGCACAGATGCGACACTTATGGGAAAGCT 1660

Query 71 TGCGCA 76

||||||

Sbjct 1661 TGCGCA 1666

**SEMA3E**

TGGAAGAATGCATAATGAAGGGAAAAGATGCGGGTGAATGTG

[ref|NM_012431.1|](http://www.ncbi.nlm.nih.gov/entrez/query.fcgi?cmd=Retrieve&db=Nucleotide&list_uids=6912649&dopt=GenBank&RID=1NHUAEX9016&log$=nuclalign&blast_rank=1) [
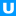
](http://www.ncbi.nlm.nih.gov/entrez/query.fcgi?db=nucleotide&cmd=Display&dopt=nucleotide_unigene&from_uid=6912649&RID=1NHUAEX9016&log$=unigenealign&blast_rank=1)[
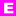
](http://www.ncbi.nlm.nih.gov/entrez/query.fcgi?db=geo&term=6912649%5Bgi%5D&RID=1NHUAEX9016&log$=geoalign&blast_rank=1)[
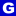
](http://www.ncbi.nlm.nih.gov/entrez/query.fcgi?db=gene&cmd=search&term=6912649%5BNUID%5D&RID=1NHUAEX9016&log$=genealign&blast_rank=1)[
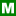
](http://www.ncbi.nlm.nih.gov/mapview/map_search.cgi?direct=on&gbgi=6912649&THE_BLAST_RID=1NHUAEX9016&log$=mapalign&blast_rank=1) Homo sapiens sema domain, immunoglobulin domain (Ig), short basic domain, secreted, (semaphorin) 3E (SEMA3E), mRNA Length=6474, [GENE ID: 9723 SEMA3E](http://www.ncbi.nlm.nih.gov/sites/entrez?db=gene&cmd=search&term=9723&RID=1NHUAEX9016&log$=geneexplicitnucl&blast_rank=1)

Score = 83.8 bits (42), Expect = 8e-15

Identities = 42/42 (100%), Gaps = 0/42 (0%)

Strand=Plus/Plus

Query 1 TGGAAGAATGCATAATGAAGGGAAAAGATGCGGGTGAATGTG 42

||||||||||||||||||||||||||||||||||||||||||

Sbjct 771 TGGAAGAATGCATAATGAAGGGAAAAGATGCGGGTGAATGTG 812

**SEMA3G**

AGGCAGCAGACGTGAGGAGTGGTTCTGGAGGAGCTCCAGGTGTTTAAGGTGCCAACACCTATCACCGAAATAATAACCTC

[ref|NM_020163.1|](http://www.ncbi.nlm.nih.gov/entrez/query.fcgi?cmd=Retrieve&db=Nucleotide&list_uids=9910361&dopt=GenBank&RID=1NJ1H2UP014&log$=nuclalign&blast_rank=1) [
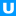
](http://www.ncbi.nlm.nih.gov/entrez/query.fcgi?db=nucleotide&cmd=Display&dopt=nucleotide_unigene&from_uid=9910361&RID=1NJ1H2UP014&log$=unigenealign&blast_rank=1)[
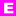
](http://www.ncbi.nlm.nih.gov/entrez/query.fcgi?db=geo&term=9910361%5Bgi%5D&RID=1NJ1H2UP014&log$=geoalign&blast_rank=1)[
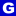
](http://www.ncbi.nlm.nih.gov/entrez/query.fcgi?db=gene&cmd=search&term=9910361%5BNUID%5D&RID=1NJ1H2UP014&log$=genealign&blast_rank=1)[
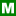
](http://www.ncbi.nlm.nih.gov/mapview/map_search.cgi?direct=on&gbgi=9910361&THE_BLAST_RID=1NJ1H2UP014&log$=mapalign&blast_rank=1) Homo sapiens sema domain, immunoglobulin domain (Ig), short basic domain, secreted, (semaphorin) 3G (SEMA3G), mRNA Length=4700, [GENE ID: 56920 SEMA3G](http://www.ncbi.nlm.nih.gov/sites/entrez?db=gene&cmd=search&term=56920&RID=1NJ1H2UP014&log$=geneexplicitnucl&blast_rank=1)

Score = 106 bits (57), Expect = 3e-21

Identities = 66/70 (94%), Gaps = 2/70 (2%)

Strand=Plus/Plus

Query 4 CAGCAG-ACGTGAGG-AGTGGTTCTGGAGGAGCTCCAGGTGTTTAAGGTGCCAACACCTA 61

|||| | || ||||| ||||||||||||||||||||||||||||||||||||||||||||

Sbjct 1421 CAGCTGAACCTGAGGAAGTGGTTCTGGAGGAGCTCCAGGTGTTTAAGGTGCCAACACCTA 1480

Query 62 TCACCGAAAT 71

||||||||||

Sbjct 1481 TCACCGAAAT 1490
