# Supplementary material for: Semaphorin, neuropilin and VEGF expression in glial tumours: SEMA3G, a prognostic marker?
Source: Br J Cancer. 2008 Sep 9;99(7):1153–60. doi: 10.1038/sj.bjc.6604641 (PMC2567090; doi:10.1038/sj.bjc.6604641)
Supplement: Supplementary Table 1 [file 6604641x3.doc]

**MD/2008/3038 revised version**

**Supplementary Table 1**

| Primer name | orientation | Primer sequences  5’ to 3’ orientation | PCR product  size in bp |
| --- | --- | --- | --- |
| GAPDH | Forward | TGCACCACCAACTGCTTAGC | 87 |
|  | Reverse | GGCATGGACTGTGGTCATGAG |  |
| VEGF | Forward | CAAGACAAGAAAATCCCTGTGG | 162 |
|  | Reverse | CCTCGGCTTGTCACATCTG |  |
| NRP1 | Forward | ATCACGTGCAGCTCAAGTGG | 167 |
|  | Reverse | TCATGCAGTGGGCAGAGTTC |  |
| NRP2 | Forward | GGATGGCATTCCACATGTTG | 152 |
|  | Reverse | ACCAGGTAGTAACGCGCAGAG |  |
| SEMA3A | Forward | AGAGACGCACAAGACGACAAGA | 89 |
|  | Reverse | GCTGTGGCCATGGTGATTATC |  |
| SEMA3B | Forward | ATGCCTACAACCGCACCCA | 81 |
|  | Reverse | GGTGGCCCACTTCCACAAAG |  |
| SEMA3C | Forward | ATCGAGTGAACGCTGCTGATG | 111 |
|  | Reverse | GCTCGCCACTGACAGAGTTGTT |  |
| SEMA3D | Forward | TGGAATTGTCTCTGAAGCAGCA | 101 |
|  | Reverse | TGCGCAAGCTTTCCCATAAG |  |
| SEMA3E | Forward | TTGGAGAGAATCAGTGACGGCT | 104 |
|  | Reverse | TTTGCACATTCACCCGCAT |  |
| SEMA3F | Forward | AGCAGACCCAGGACGTGAG | 114 |
|  | Reverse | AAGACCATGCGAATATCAGCC |  |
| SEMA3G | Forward | GCTCAAAGTCATCGCTCTCCAG | 103 |
|  | Reverse | CATTTCGGTGATAGGTGTTGGC |  |
| SEMA4D | Forward | CCCACATCCACGAGGTGG | 100 |
|  | Reverse | CGCACTCCCATCTCAGCTCT |  |

SEMA3F and GAPDH primers were previously validated (Brambilla *et al*, 2000) as NRP1, NRP2 and VEGF primers (Lantuéjoul *et al*, 2003).
