# Supplementary material for: Semaphorin, neuropilin and VEGF expression in glial tumours: SEMA3G, a prognostic marker?
Source: Br J Cancer. 2008 Sep 9;99(7):1153–60. doi: 10.1038/sj.bjc.6604641 (PMC2567090; doi:10.1038/sj.bjc.6604641)
Supplement: Supplementary Table 2 [file 6604641x4.doc]

**MD/2008/3038 revised version**

**Supplementary Table 2**

A

|  | **Female gliomas**  Medians (min-max )  n=14 | **Male gliomas**  Medians (min-max)  n=24 | **Mann-Witney U test**  **(p value)** |
| --- | --- | --- | --- |
| **SEMA3A** | 0.91 (0.04-11.24) | 0.48 (0.01-18.48) | 0.967 |
| **SEMA3B** | 2.97 (0.88- 24.65) | 4.55 (0.90-17.11) | 0.740 |
| **SEMA3C** | **0.62 (0.21- 5.79)** | **1.55 (0.02-20.63)** | **0.041** |
| **SEMA3D** | 0.01 (0.00-5.49) | 0.04 (0.00-3.74) | 0.654 |
| **SEMA3E** | 0.29 (0.10-10.97) | 2.27 (0.03-130.67) | 0.074 |
| **SEMA3F** | **0.83 (0.01-3.99)** | **1.98 (0.21-36.91)** | **0.021** |
| **SEMA3G** | 0.30 (0.01-3.08) | 0.22 (0.01-4.79) | 0.562 |
| **SEMA4D** | 1.73 (0.58-4.07) | 3.20 (0.96-8.69) | 0.117 |
| **VEGF** | 46.10 (5.56-188.16) | 59.64 (5.12-287.17) | 0.481 |
| **NRP1** | 6.17 (0.67-13.98) | 6.17 (0.67-13.98) | 0.602 |
| **NRP2** | 5.93 (0.98- 16.75) | 5.93 (0.98-16.75) | 0.154 |

B

|  | **L Low-grade gliomas**  Medians (min-max ) n=11 | **High-grade gliomas**  Medians (min-max)  n=27 | **Mann-Witney U test**  **(p value)** |
| --- | --- | --- | --- |
| **SEMA3A** | 2.33 (0.27-18.48) | 0.48 (0.01-11.63) | 0.211 |
| **SEMA3B** | 4.55 (0.90-8.83) | 3.83 (0.88-24.65) | 0.786 |
| **SEMA3C** | 1.38 (0.14-5.59) | 1.34 (0.02-20.63) | 0.735 |
| **SEMA3D** | **0.59 (0.01-3.74)** | **0.01 (0.00-5.49)** | **0.035** |
| **SEMA3E** | 2.47 (0.05-130.67) | 1.40 (0.03-27.64) | 0.870 |
| **SEMA3F** | 1.34 (0.15-3.83) | 1.62 (0.01-36.91) | 0.241 |
| **SEMA3G** | 0.52 (0.07-1.28) | 0.20 (0.01-4.79) | 0.211 |
| **SEMA4D** | 3.65 (1.61-6.59) | 1.89 (0.58-8.69) | 0.155 |
| **VEGF** | **15.51 (5.56-231.65)** | **68.87 (5.12-287.17)** | **0.035** |
| **NRP1** | 8.26 (1.91-22.41) | 5.76 (0.67-26.28) | 0.218 |
| **NRP2** | 8.67 (1.36-16.75) | 6.46 (0.79-59.54) | 0.362 |

The results (medians and minims-maxims) are expressed in [2^−Δ^Ct x 1000] where ΔCt = Ct_target_ -Ct_GAPDH_
